# Supplementary figures and images for: The Impact of PCSK9 on Diabetic Cardiomyopathy: Mechanisms and Implications
Source: Biomolecules. 2025 Aug 27;15(9):1240. doi: 10.3390/biom15091240 (PMC12467878; doi:10.3390/biom15091240)

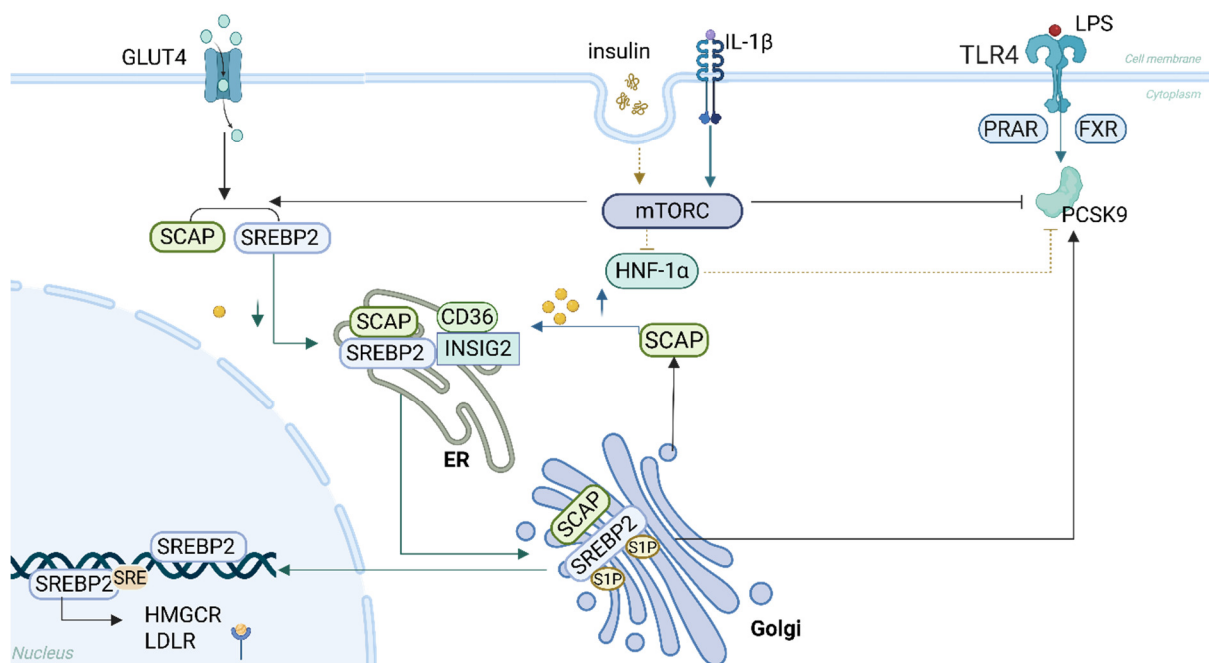

**Figure S1.** Recent advances in PCSK9-mediated regulation of lipid metabolism.

Supplement: Supplementary file 1 [file biomolecules-15-01240-s001.zip › biomolecules-3713688-supplementary.pdf]
